# 3D-printing of the elbow in complex posttraumatic elbow-stiffness for preoperative planning, surgery-simulation and postoperative control

**DOI:** 10.1186/s41205-023-00191-x

**Published:** 2023-10-06

**Authors:** Ronny Grunert, Dirk Winkler, Franziska Frank, Robert Moebius, Fabian Kropla, Juergen Meixensberger, Pierre Hepp, Maria Elze

**Affiliations:** 1 Department of Neurosurgery, University Leipzig, Liebigstr. 20, Leipzig, 04103 Germany; 2 Fraunhofer Institute for Machine Tools and Forming Technology, Theodor-Koerner-Allee 6, Zittau, 02763 Germany; 3 Department Orthopedics, Trauma Surgery and Plastic Surgery, University Leipzig, Liebigstr. 20, Leipzig, 04103 Germany

**Keywords:** Joint surgery, Elbow stiffness, Patient-specific models, 3D-printing, Preoperative planning, Postoperative control

## Abstract

**Background:**

Restoration of mobility of the elbow after post-traumatic elbow stiffening due to osteophytes is often a problem.

**Methods:**

The anatomical structures were segmented within the CT-scan. Afterwards, the Multi Jet Fusion 3D-printing was applied to create the model made of biocompatible and steam-sterilizable plastic. Preoperative simulation of osteophyte resection at the 3D-model was performed as well as the direct comparison with the patient anatomy intraoperatively.

**Results:**

The patient-specific was very helpful for the preoperative simulation of the resection of elbow osteophytes. The 3D anatomical representation improved the preoperative plan its implementation. A high degree of fidelity was found between the 3D Printed Anatomical representation and the actual joint pathology.

**Conclusions:**

Arthrolysis of complex post-traumatic bony changes is an important indication for the use of 3D models for preoperative planning. Due to the use of 3D printing and software simulation, accurate resection planning is feasible and residual bony stiffening can be avoided. 3D printing models can lead to an improvement in surgical quality.

## Background

Treating complex posttraumatic stiffness of the elbow is a major challenge due to its complex anatomy, limited accessibility and highly variable injury pattern. Post-traumatic elbow stiffness are classified as intrinsic, extrinsic, or mixed [1]. In practice, causes of post-traumatic elbow stiffness are frequently mixed. On one hand, these conditions encompass a variety of intrinsic factors such as articular incongruities, impinging osteophytes, and intra-articular adhesions that originate within the joint itself. On the other hand, they involve extrinsic factors such as capsular contractures, heterotopic ossification, and impinging hardware that occur outside the joint [2].

While adhesions and capsular contractures can often be addressed through arthroscopic or open soft-tissue release and arthrolysis, managing incongruities and impingement resulting from osteophytes or heterotopic ossification can be more challenging due to non-extensile surgical approaches that often result from lack of proper pre-op planning or potential misinterpretation of the underlying cause of stiffness.

Arthroscopic arthrolysis impresses with a lower complication and revision rates, whereby a high level of expertise in elbow arthroscopy is required. Common complications and revision reasons were documented with repeat stiffness or nerve symptoms. Distraction arthrolysis is another surgical method, but it is technically challenging and only suitable for a limited number of patients. and arthrolysis, or distraction arthrolysis. Interposition arthroplasty can be an additional treatment option to improve function and proved pain relief in younger patients to avoid joint replacement in case of posttraumatic osteoarthritis.

Accurate analysis of imaging plays a crucial role in achieving successful outcomes. In this regard, preoperative simulation can provide valuable support in determining the appropriate surgical approach and intraoperative strategy.

The management of incongruities and conflicts caused by osteophytes or heterotopic osteophytes may be limited by either the surgical approach or a misinterpretation of the key element causing the restricted range of motion.Elbow arthrolysis requires careful preoperative analysis and planning to achieve satisfactory results. Compromises must be avoided to maintain the best possible elbow function and patient satisfaction. However, the practical procedure shows in particular that post-traumatic 3D malformations are not sufficiently shown and recorded despite visual 3D reconstructions of regularly used CT analyses. Surgeons apply 3D-printing technology for nearly all areas of orthopedic trauma surgery and elbow [3, 4]. Haptic 3D models can be created from standard CT datasets as an additional tool, enable the surgeon to plan surgery more precisely. Post-traumatic bony changes regularly show extra-anatomical callus formation [3], secondary osteophyte formations, fracture consolidation in malposition after incomplete reduction or in children with subsequent malgrowth a 3-dimensionally altered anatomy [5, 6]. These lead to incongruence of the joint partners and cause mechanical movement restrictions. Particularly small osteophytes can be easily detected and removed both CT-morphologically and intraoperatively visually. However, pronounced osteophytes, callus formations or misconsolidated fractures with misaligned axes can only be analyzed to a limited extent and are not always adequately addressed in surgical planning.

### Surgical principle and objective

The aim of the present procedure was the preoperative simulation of the resection of elbow osteophytes with the help of a previously created 3D-print. The 3D-model intended to serve the surgeon as a strategy and decision-making aid. In addition, it may serve as control while performing intraoperative maneuvers and to postoperatively compare the final results with the intended surgical plan.

## Methods

### Indications

**Fig. 1:**
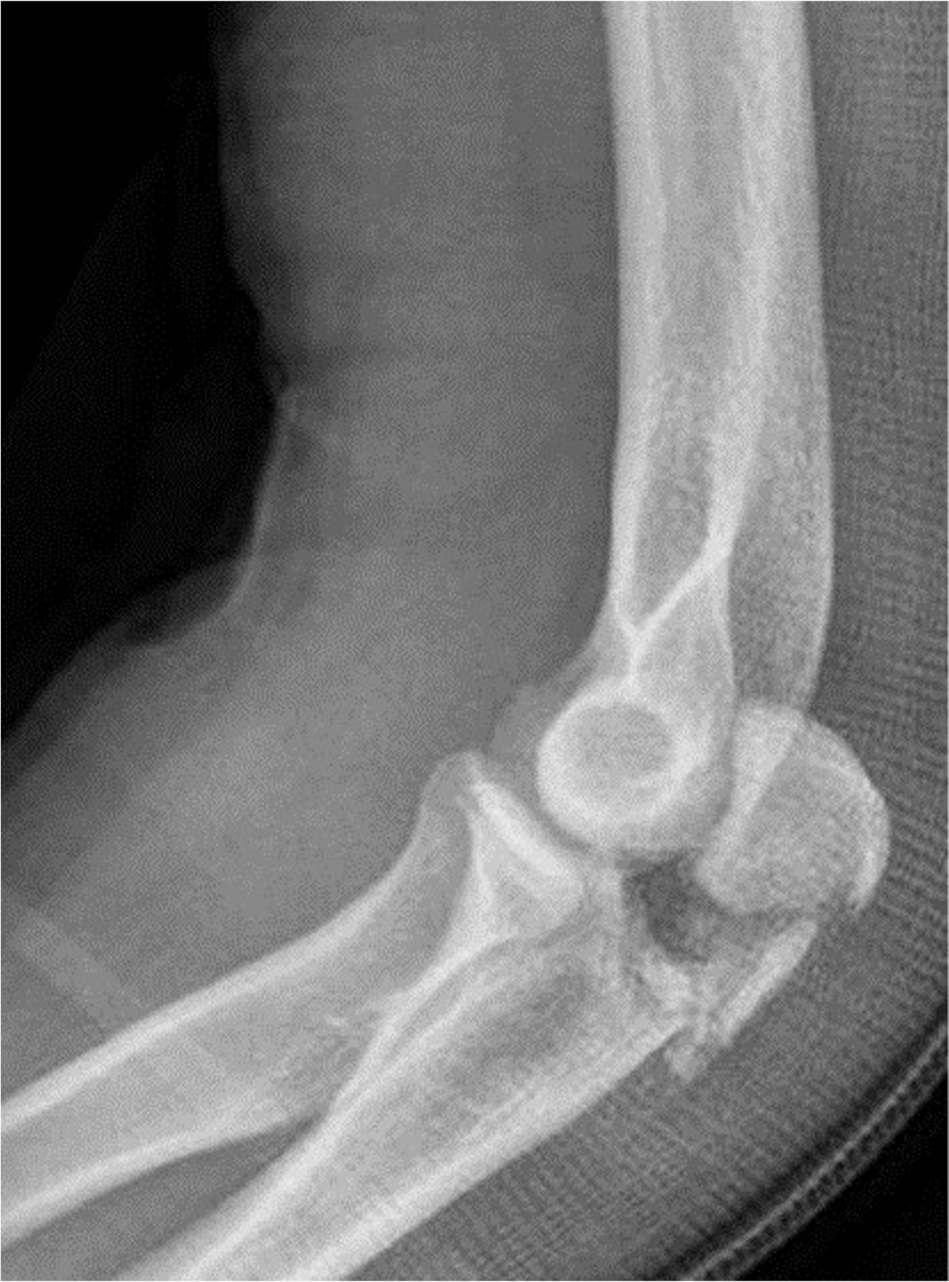
Preoperative lateral radiograph of an elbow with an olecranon fracture and comminuted articular surface

The patient was 38 years old with a posttraumatic elbow stiffness with relevant osseous pathologies e.g. osteophytes, and ossifications. After a fall from 10 m height the polytrauma injured patient suffered a severe forarm fracture dislocation, which is called a Monteggia fracture, 1,5 years before. The injury is a combination of a proximal ulna fracture and a radial head dislocation (Fig. 1).

**Fig. 2:**
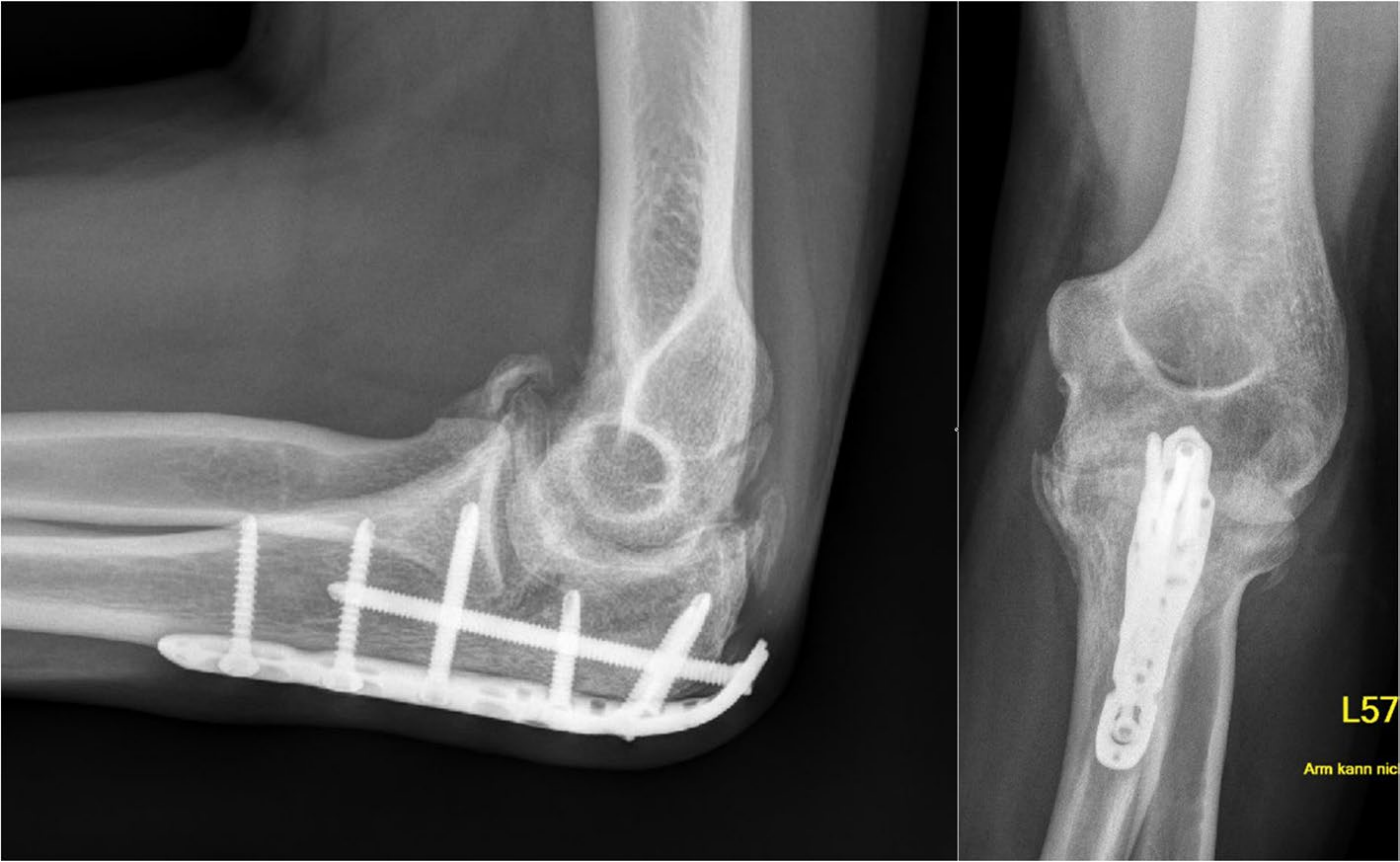
Radiographs in lateral and a.p. view of the left elbow of a 38 year old patient. One year after sustaining a Monteggia fracture and plate osteosynthesis of the olecranon

The therapy of choice contained a plate osteosynthesis of the olecranon and repositioning of the radial head. In the course of this, a restriction of movement became apparent. One year after trauma the range of motion stopped by extension/flexion arc 0/40/135°, despite intensive physiotherapy. Radiographs showed a healed olecranon fracture with massive osteophyts and heterotrophic ossifications in lateral and a.p. view (Fig. 2). A CT-scan was initiated to determine the extent of osteophyts, their positions and to rule out a 3-dimensional malformation of the articular surface of the olecranon. The CT scan confirmed the healed olecranon fracture without gross malformation but also showed radially and ulnarly projecting osteophytes. However, the osteophyte most impairing movement could not be identified with this imaging. So we decided to make a 3D-model to analyze the osseous key structures of stiffness.

### Segmentation of CT-data and 3D-printing

**Fig. 3:**
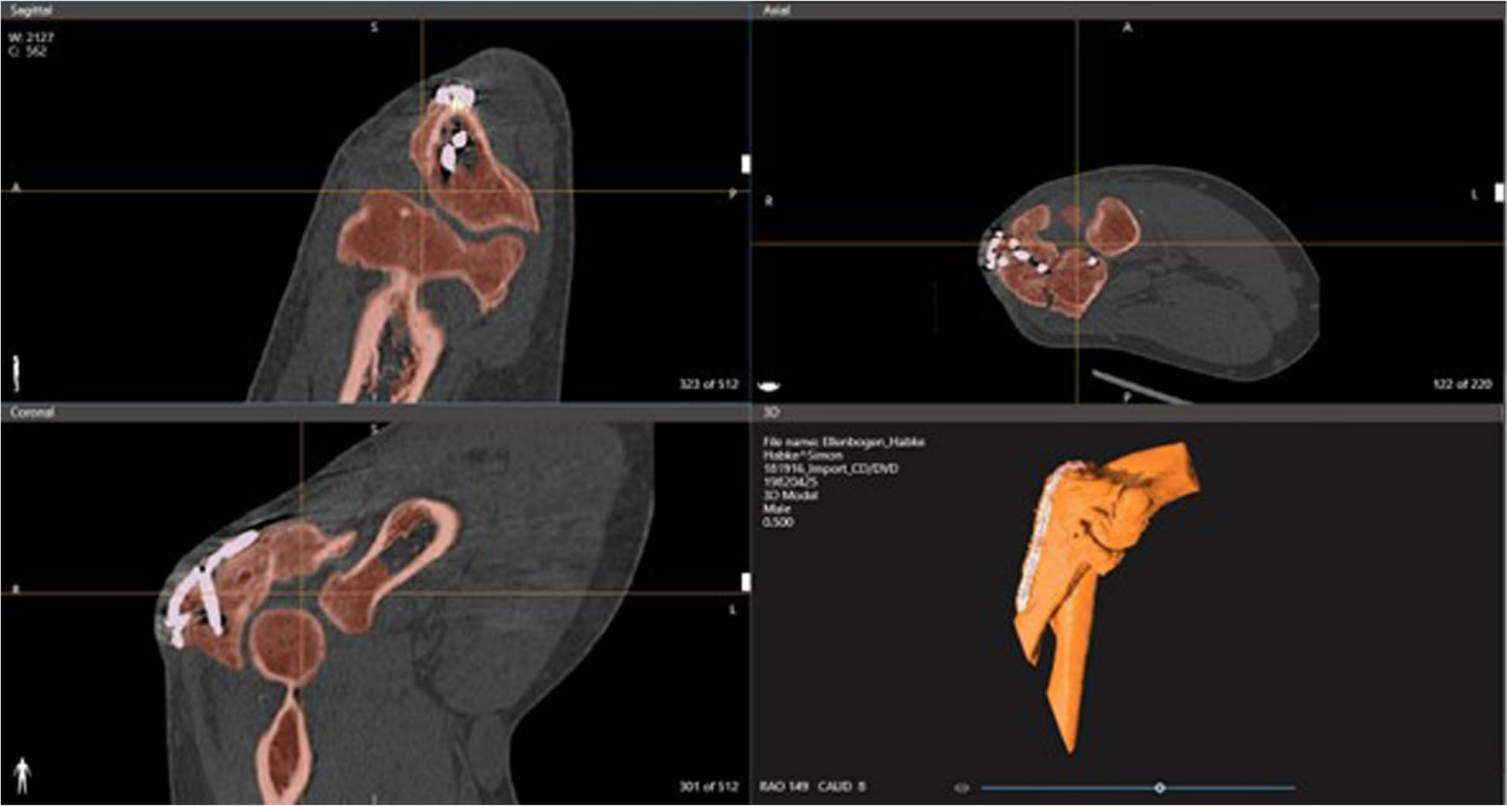
Segmentation of the anatomical structures with the help of CT-images

**Fig. 4:**
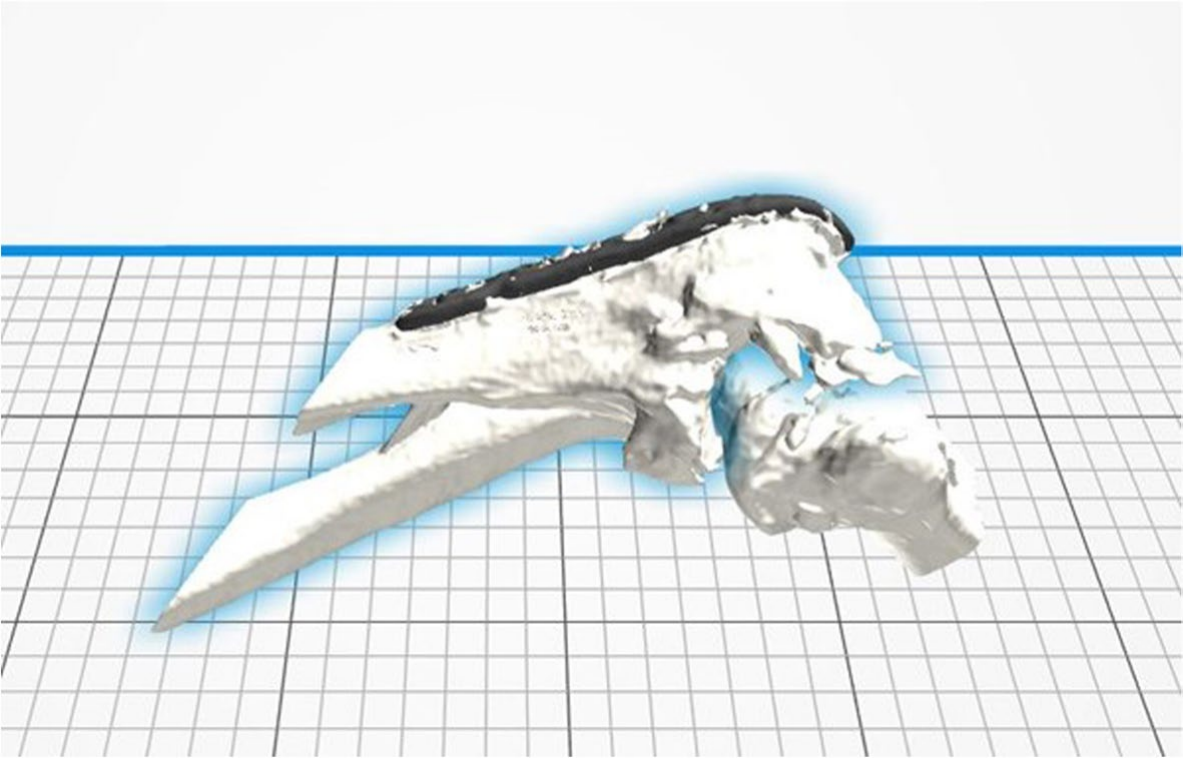
Virtual 3D model positioned in the 3D-printing software

The patient was scanned with a Multispiral CT with secondary reconstruction of 1 mm slice thickness with Helix CT (Philips Ingenuity Core, Hamburg, Germany) according to the standard hospital protocol using a bone core for sharp visualization of the bone fragment edges. Subsequently, the regions of interests were segmented in the CT-images (Fig. 3) by biomedical engineers with the software D2P™ (3D Systems Inc., Rock Hill, USA). The accuracy of the segmentation was controlled by an experienced joint surgeon. After the final check, a 3D-mesh of the elbow joint was calculated and exported as a standard tessellating language file (STL) which was transferred to the 3D-printer Multi Jet Fusion 580 Color (Hewlett-Packard Inc., Palo Alto, USA) of the clinic's internal 3D printing research laboratory (Fig. 4).

The patient-individual model was printed with the biocompatible material polyamide (PA). After the 3D-print the model has been subjected to post-processing. Glass bead blasting of the model surface was performed to remove loose powder particles with the SMG 50 Rapid Ex (MHG GmbH, Duesseldorf, Germany).

As final step before surgery, the 3D-printed model was 20 min. steam sterilized with 130 °C in the central sterilization department of the University Hospital Leipzig.

### Preoperative planning

**Fig. 5:**
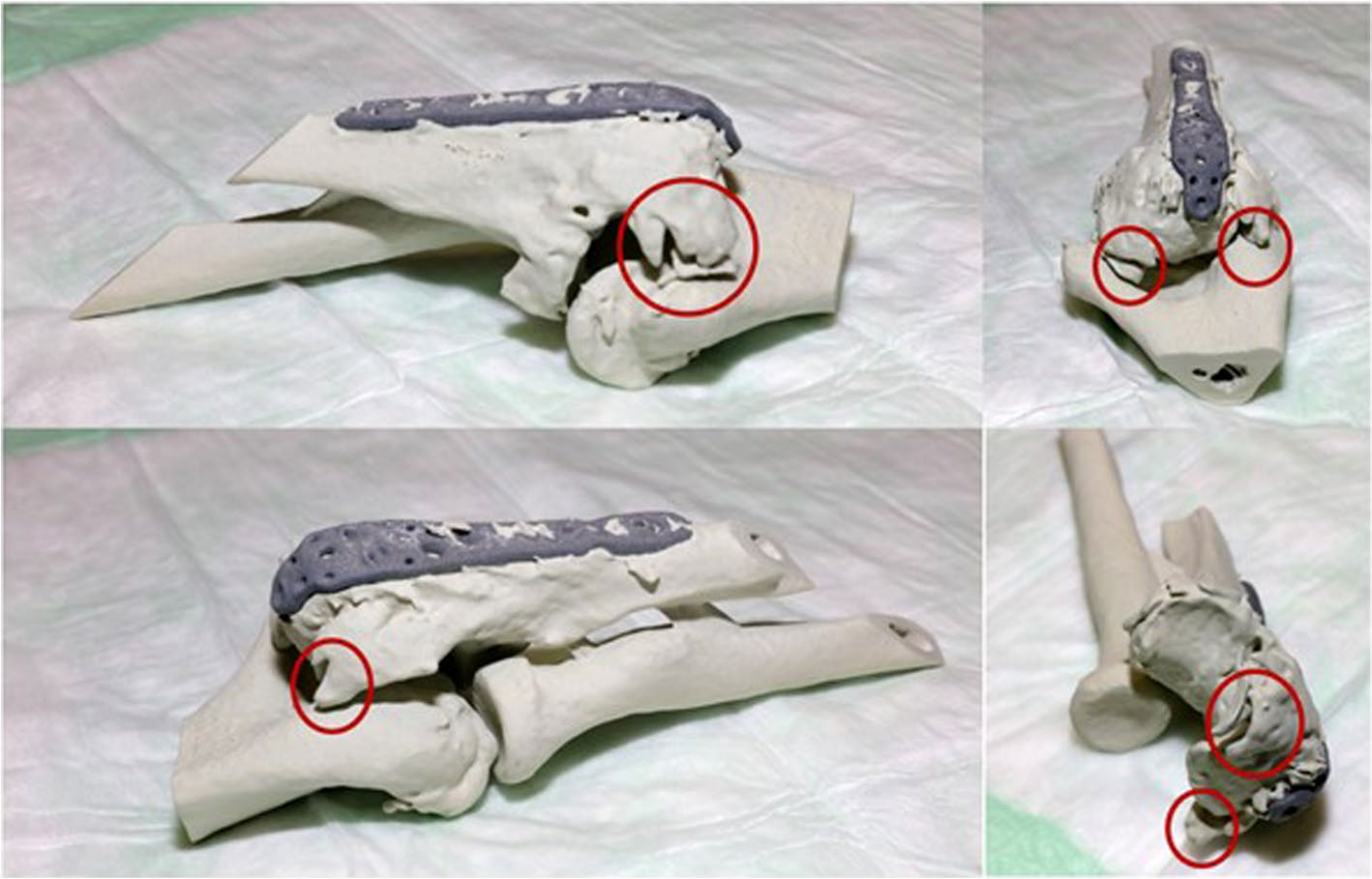
Preoperative simulation of the surgery

**Fig. 6:**
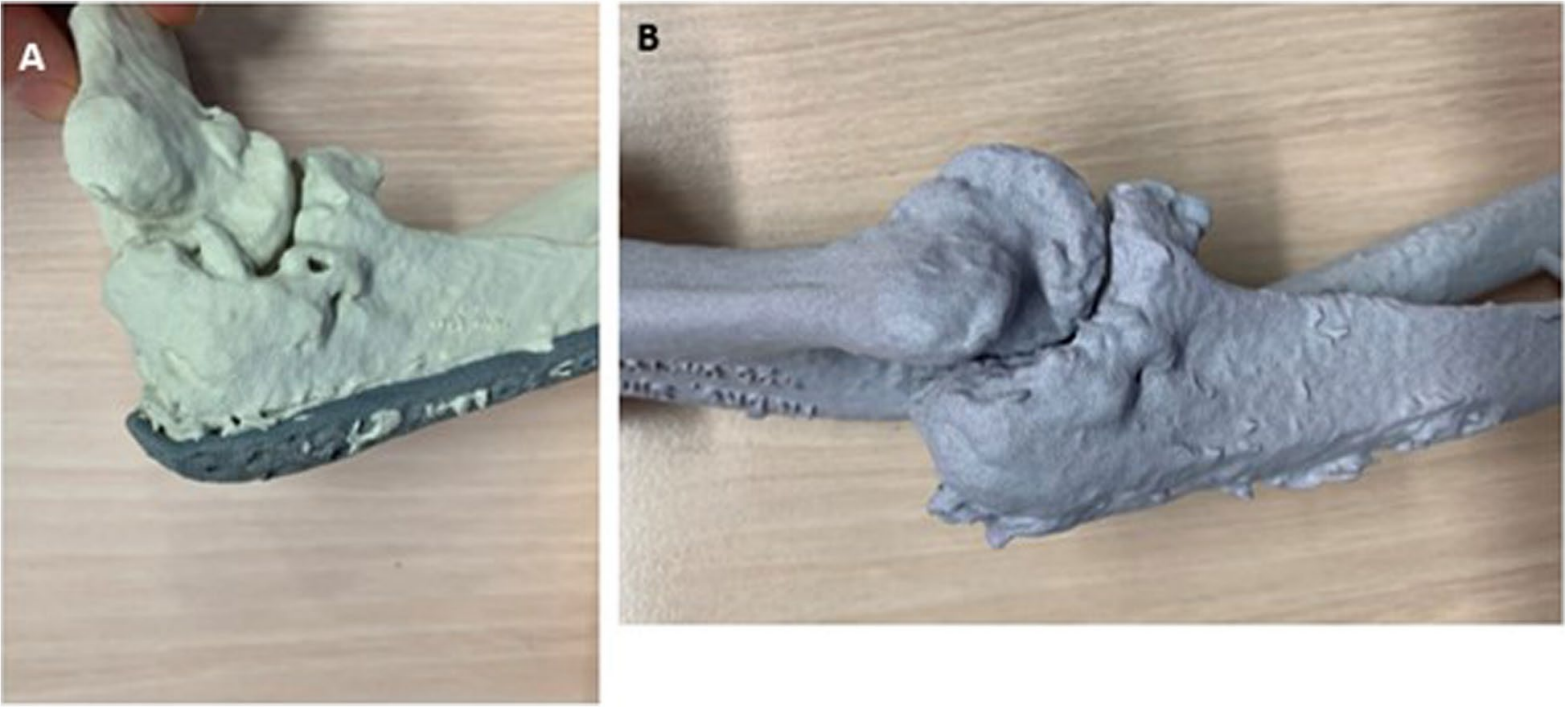
**A** Preoperative situation with extension deficit. **B **Postoperative situation with increased extension of the model

As first step, the surgeon (Figs. 5 and 6) assessed the plausibility of the 3D print. Based on the CT scan, it was to be expected that the ulnar osteophytes could be the key structure impeding movement. Subsequently the surgery was simulated direct at the model and the osteophytes were removed with chisels. However, the simulation did not show any improvement in movement. Therefore, the resection was continued for the next most likely radial osteophytes step-by-step until better movement was achieved. The 3D model simulation showed preoperatively that the radial osteophytes are the key structures in bony motion stiffness. Despite everything, free mobility could not be restored with the bony resections. Finally, the specification of the surgical approach, order and relevance of resection as surgical strategy could be determined and transferred to the patient. The patient could be informed about the realistically expected surgical result.

### Intraoperative situation

**Fig. 7:**
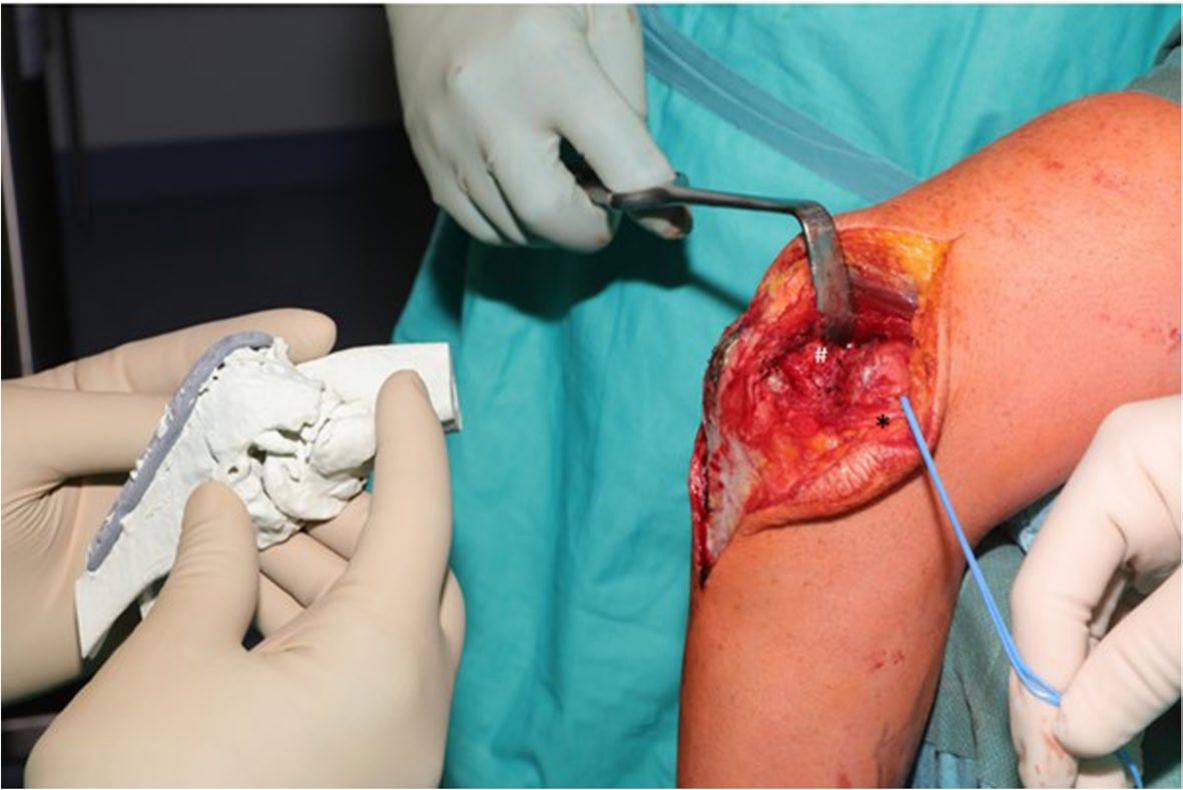
Intraoperative comparison of the patient-specific 3D-model with the anatomical structures of the real patient

**Fig. 8:**
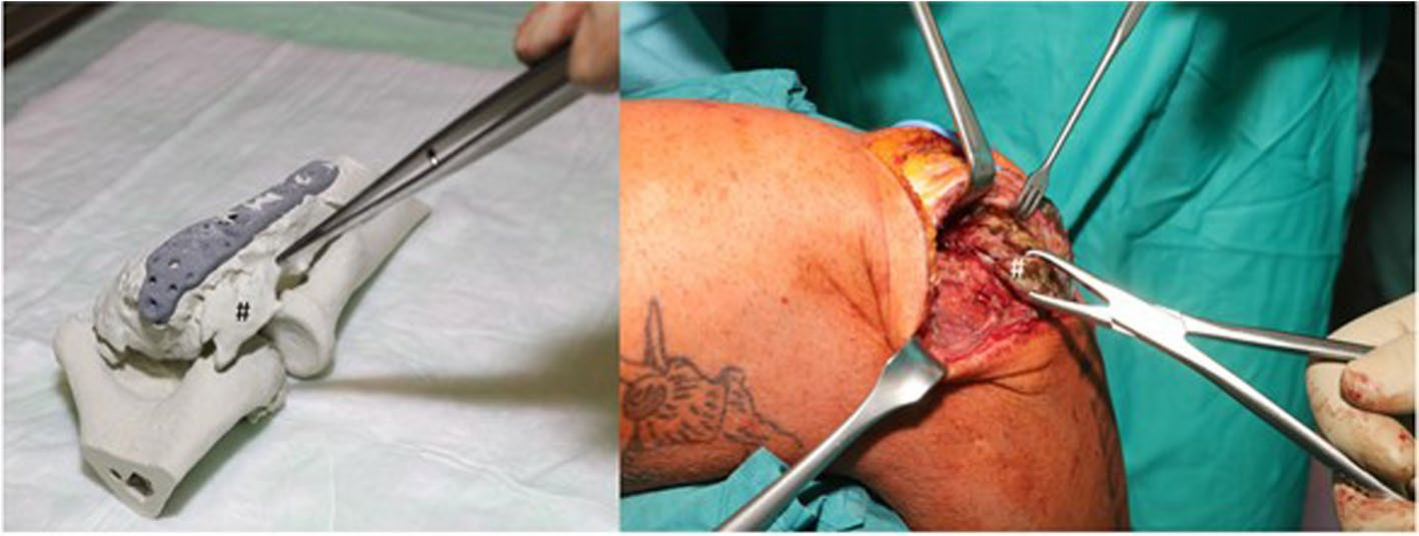
Intraoperative representation of the radial osteophytes at the 3D-model and the patient

Due to the existing dorsal scar, the dorsal paratricipital approach was used again. Based on the preoperative simulation, all osteophytes and callus formations could be addressed via this approach. This is also the standard approach to neurolysis of the ulnar nerve, which had to be identified and protected (Fig. 7). The implant was removed except for one screw, which was left. This was broken and could only have been removed by damaging the bone. The osseous structures were compared with the existing 3D print model, which showed a realistic matching. It followed a successive removal of the supposedly disturbing ulnar osteophytes. The interim balance does not show any significant improvement in the extension, which was to be expected based on the preoperative planning. However, the resection of the ulnar osteophytes was carried out to avoid ulnar neuropathy due to mechanical irritation. The radial osteophytes are also visualized and the osteophytes are successively removed from the olecranon towards the distal end (Fig. 8). Immediately the progress was checked by passive extension and flexion of the elbow joint and compared to the 3D model. The movement stop was softer, but the simulated freedom of movement could not be achieved. Therefore, extensive ventral and dorsal arthrolysis was performed. Adhesions were resected from the fossa olecrani. Besides that, an extensive ventral capsule thickening was addressed by capsulotomy. The range of motion showed an extension/flexion arc of 0/20/130°.

### Postoperative management

Regional catheter analgesia supported the inpatient intensive physiotherapy until the intraoperative range of motion was reached. Passive-assisted and passive-continuous movement was performed to avoid new adhesions daily.. After discharge, the independent exercise with the passive continuous motion machine and active exercise with the physiotherapist were continued. Nonselective or selective NSAIDs for preventing heterotopic ossification were applied [7, 8].

## Results

**Fig. 9:**
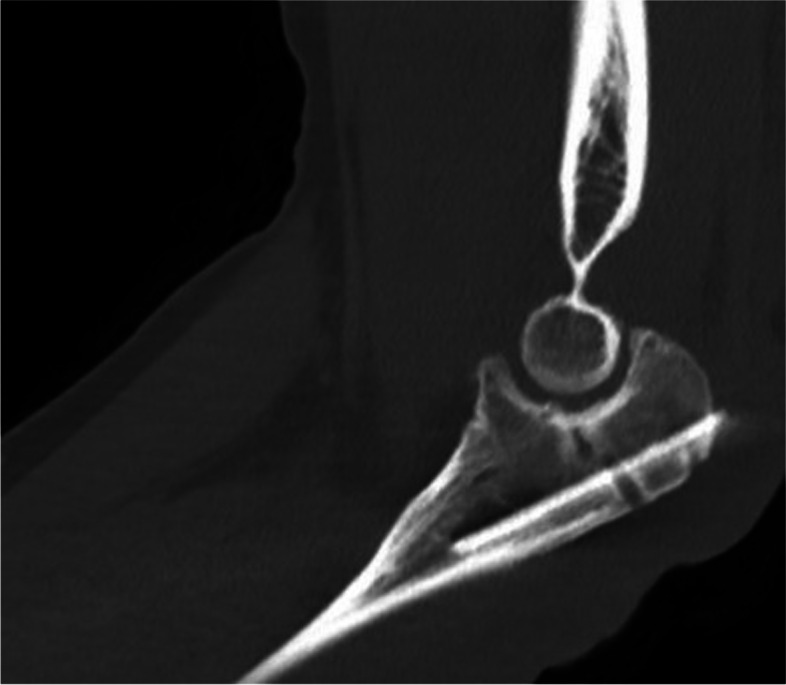
Postoperative CT-scan showing the centered joint, the remaining screw and screw holes after hardware removal

A postoperative CT scan showed the centered joint, confirmed the remaining screw and complete resection of the interfering osteophytes (Fig. 9). The planned simulation on the 3D model could be transferred to the patient. The renewed analysis of the greater sigmoid notch suggests that a marginal reduction was caused by the initial osteosynthesis, which explains the residual extension deficit. These small osseous malformations can cause significant movement restrictions in a delicate joint.

**Fig. 10:**
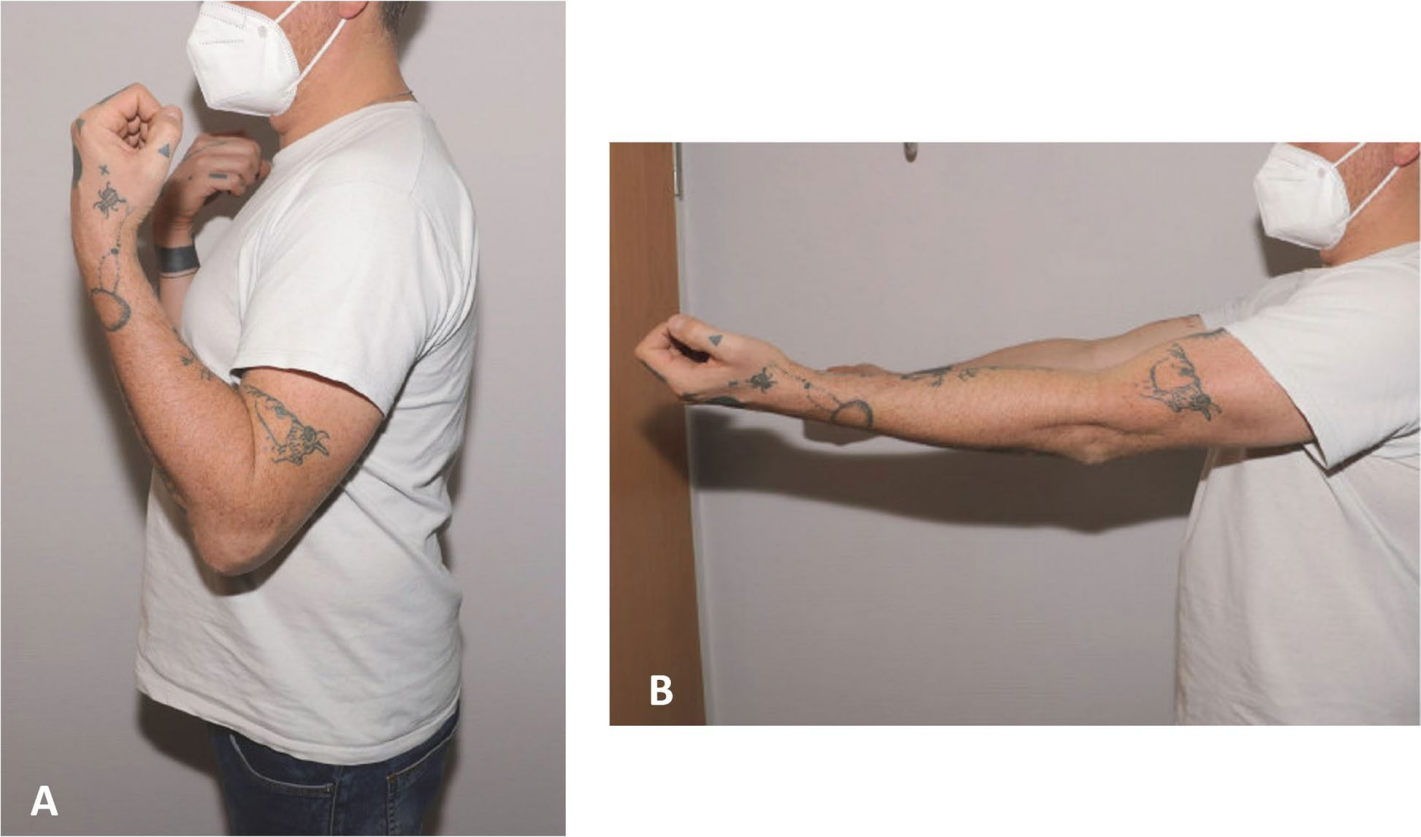
The patient shows a residual slight flexion (**A**) ad extension (**B**) deficit in side comparison

One year after revision surgery, the patient was very satisfied and was able to demonstrate intraoperative mobility by an extension/flexion arc 0/20/130° (Fig. 10).

## Discussion

**Fig. 11:**
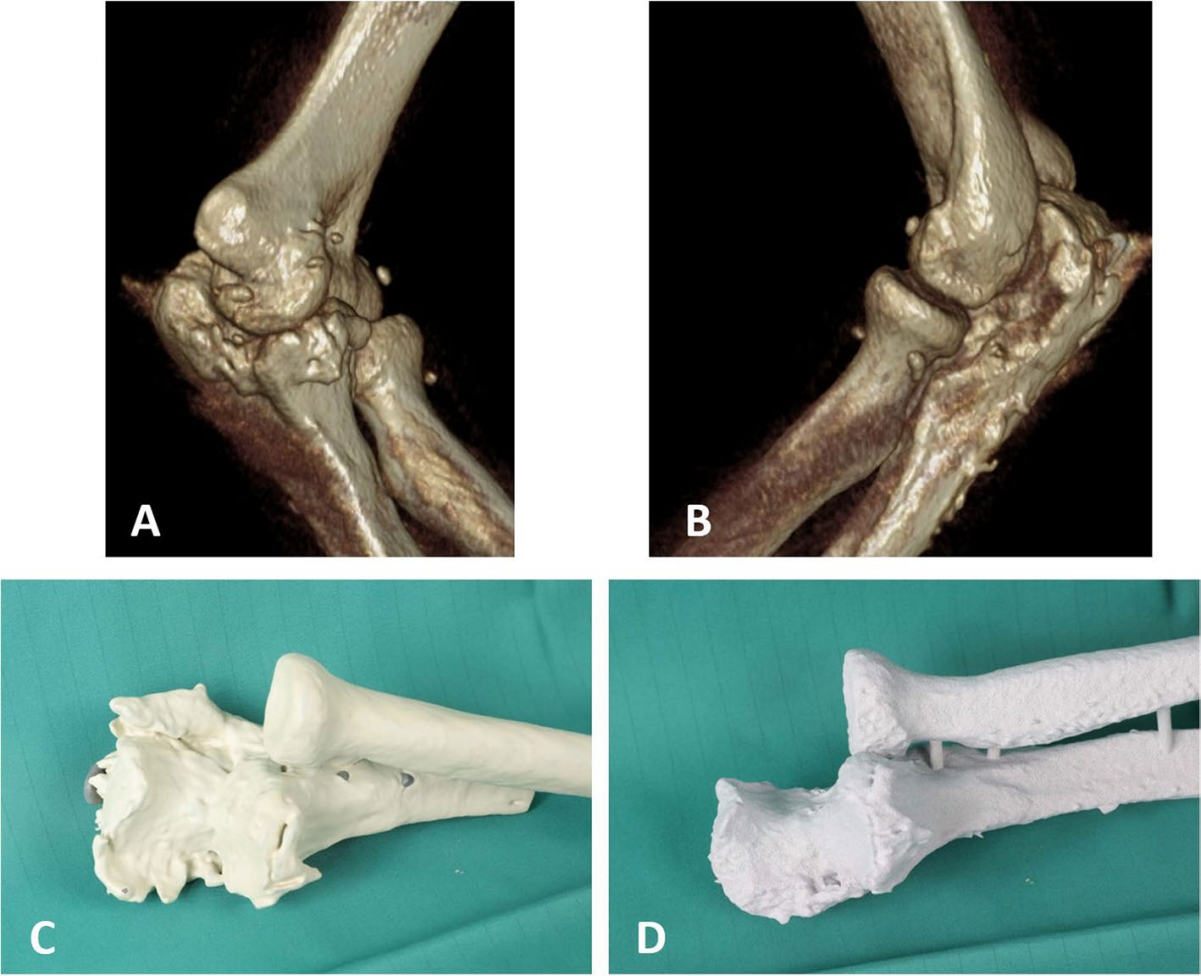
Postoperative CT scan (**A**, **B**) and 3D-model confirming the resection of all interfering osterophytes (**C**, **D**)

The Preoperative review of the 3D-model allowed the surgeon to anticipate intraoperative difficulties, select optimal surgical approach, plan implant placement and access the need for special equipment. Finally, it could help to evaluate the restoration of the individual anatomy (Fig. 11). However, the intraoperatively detected 3D deformity cannot always be corrected after soft-tissue and bony arthrolysis of callus and osteophytes without adequate preoperative planning. Depending on the extent, the therapy of the malformation or misalignment can range from coplaning to osteotomies and re-osteosynthesis, which cannot and should not be implemented in the surgical setting ad hoc. Haptic 3D-printed models offer the surgeon a practical way of planning the step-by-step preoperative correction and success control of the achieved range of motion, which cannot be achieved with a pure 3-dimensional representation of CT data sets. Without haptic or computer-based movement simulation, the relevant bony key component of the functional disability can be misinterpreted. Li et al. showed that when comparing 3D printed models, simulated 3D reconstructions and two-dimensional CT slice examinations, complex anatomy could best be understood and analyzed using 3D printed models [9]. The 3D model gave the user a haptic experience of the pathology to be treated and allowed the patient to get an idea of the surgical performance.The extent of complex malformations can be underestimated in 3D CT reconstructions. Furthermore, haptic models offer a possibility of improved patient communication, so that the anatomical pathology, the surgical procedure, the risk of complications as well as expectations and realistic surgical goals can be made understandable to the patient preoperatively [10]. Patients showed increased compliance to postoperative rehabilitation measures and improved empowerment. Compared to conventional surgical planning based on two-dimensional image data sets, preoperative surgical planning on fractured 3D models has already shown a shorter surgical time, reduced blood loss and improved elbow function in the outcome [10–13]. An intraoperative radiation reduction for patients and surgical team was achieved through more detailed preoperative planning [11, 12]. The relationship between virtual simulation programs and haptic 3D printing models in the planning of anatomically complex operations has not yet been sufficiently documented. In addition, there is no comprehensive access to alternative computer software that would enable surgery simulation with resections and subsequent movement testing.

Although the contribution of the engineers who affect image processing and 3D printing is made outside of the clinical routine, the application and implementation of 3D printing technology requires constant and critical supervision by an experienced surgeon. Additionally, the 3D printing technology is associated with additional costs for the material used, including equipment maintenance, and reduces the revenue situation for the treating facility. As a consequence of its surgical value and not inconsiderable additional effort, the use of 3D-printing in the field of surgery must currently be assessed critically, and these options are certainly only reserved for selected questions and applications. It is in everyone's interest to help this very valuable technology achieve a breakthrough through growing routines and further optimization of process flows. We believe that using modern 3D printers will greatly simplify the process of making custom models and that this technology may be more widely used in the future.

## Conclusions

Arthrolysis with addressing of complex post-traumatic bony changes represents an important indication for preoperative extended planning using 3D models. Precise resection planning can be carried out with both 3D printing and software simulation and residual bony stiffness can be avoided. Furthermore, in addition to increased patient satisfaction through more understandable surgical information, haptic models offer surgeons the opportunity to reduce learning curves and intraoperative complications, to improve theoretical knowledge and to train practical skills. 3D printed models can lead to an improvement in surgical quality.

## Data Availability

The data that support the findings of this study are available from the corresponding author, upon request.
